# Using Mathematical and Statistical Analysis to Investigate the Correlation between Exacerbation of Chronic Obstructive Pulmonary Disease and Risk of Subclinical Atherosclerosis

**DOI:** 10.3390/diagnostics13040623

**Published:** 2023-02-08

**Authors:** Xiaochun Dai, Jiahui Chen, Lili Shao

**Affiliations:** The Affiliated Kangning Hospital of Wenzhou Medical University, Wenzhou 325000, China

**Keywords:** carotid artery intima-media thickness, chronic obstructive pulmonary disease, cardiovascular risk, ankle-brachial index, atherosclerosis

## Abstract

**Purpose:** As the number of patients with chronic obstructive pulmonary disease continues to increase, it is increasingly important to understand the impact of cardiovascular risk on the progression of chronic obstructive pulmonary disease, which can provide guidance for clinical medication and recommendations for patient care and rehabilitation. The purpose of this study was to investigate the relationship between cardiovascular risk and the progression of chronic obstructive pulmonary disease (COPD). **Methods:** Selected COPD patients admitted to hospital from June 2018 to July 2020 were included in the study for prospective analysis, and patients who showed more than two instances of moderate deterioration or severe deterioration within one year before the consultation were defined as COPD patients, and all participants underwent relevant tests and assessments. **Results:** Multivariate correction analysis showed that a worsening phenotype improved the risk of carotid artery intima-media thickness exceeding 75% by nearly three times, and it had no relation with the degree of COPD severity and global cardiovascular risk; in addition, the relationship between a worsening phenotype and high carotid intima-media thickness (c-IMT) was more pronounced in patients under 65 years of age. **Conclusions:** The existence of subclinical atherosclerosis is individually related to the worsening phenotype, and the difference is more obvious in young patients. Therefore, the control of vascular risk factors in these patients should be strengthened.

## 1. Introduction

Patients with an acute aggravation of chronic obstructive pulmonary disease (AECOPD) typically die from cardiovascular disease. Studies have shown that carotid atherosclerosis is associated with the severity of chronic obstructive pulmonary disease (COPD) [1,2,3]. According to a study, the prevalence of COPD among Chinese adults over 40 years of age is as high as 8.2%, which has become an important disease factor threatening people’s health. The clinical research of COPD has also received great attention from the respiratory academic circle. COPD exacerbation is defined as a daily change in a patient’s condition from a steady state to a persistent deterioration beyond normal [4]. Exacerbations are traditionally understood as temporary clinical decompensation; its consequences are constrained to the time frame of the event itself. Recent investigations have shown that many patients do not fully recover following exacerbations, which are followed by pulmonary and systemic repercussions; these patients experienced the rapid worsening of lung function, including deterioration of the BODE index, and increased incidence of cardiovascular diseases such as myocardial infarction [3,4]. Despite its drawbacks, the global assessment of cardiovascular risk (CVR) is regarded as the best method for predicting and preventing cardiovascular disease [5]. In addition, recent research has revealed that c-IMT can be used to predict cardiovascular events and is significantly associated with bronchial obstruction and other pulmonary function parameters [6,7]. The ankle-brachial index (ABI) is a straightforward, practical, and affordable technique that was originally widely used to detect peripheral arterial disease, and the use of ABI as a measure of subclinical atherosclerosis is supported by recent evidence [8]. Currently [8,9,10], few studies have compared CVR and subclinical atherosclerosis with COPD; therefore, this study’s objective was to better stratify COPD patients’ prognoses by examining the relationship between vascular risk and COPD exacerbations.

## 2. Materials and Methods

### 2.1. Research Object

Selected COPD patients who visited our hospital from June 2018 to July 2020 were included in the study for prospective analysis. Patients with a history of COPD who had uncontrolled dyspnea or exacerbations even with medical handling, or who had progressive illness necessitating further treatments, including oxygen therapy, or consideration of lung transplantation, which were excluded. The diagnosis of COPD was made according to the treatment guidelines. The diagnosis of coronary artery atherosclerosis was based on the clinical diagnostic criteria of coronary artery atherosclerotic heart disease in the first National Academic Conference of Internal Medicine of Chinese Medical Association in 1980. **Inclusion criteria:** ① Diagnosed as AECOPD; ② Age ≥ 18 years; ③ AECOPD diagnosis in accordance with the 2017 GOLD guidelines. **Exclusion criteria:** ① Combined with hyperblood pressure, hyperlipidemia, diabetes, and rheumatic immune diseases; ② Combined with blood fluid disease; ③ Patients with tumors and other lung diseases; ④ Liver function not complete. Age and gender did not significantly differ between the two groups (*p* > 0.05). The study was approved by the hospital ethics committee.

### 2.2. Method

(1) A unified registration form was used to collect basic information of the subjects, including age, gender, chronic medical history (hypertension, diabetes), tobacco and alcohol history, etc. (2) Biochemical indexes: Fasting venous blood was collected, and routine biochemical indexes such as blood lipids were detected using a biochemical analysis system. (3) Serum ultra-sensitive C-reactive protein was detected using an immunoassay system. (4) We used the Framingham Global Cardiovascular Risk Score [10], SCORE Risk Chart [10], SCORE-HDL [11], Regicor [12], and the COPD Coronary Disease Risk Assessment Scale [13] (COPD CoRi) to assess the risk of CVR. (5) All patients underwent spirometry and bronchodilator testing using two doses of terbutaline and the Turbuhaler^®^ system in accordance with SEPAR guidelines [14]. (6) Participants were given a 6-min walk test according to the guidelines [15]. (7) Carotid artery IMT measurement with echo Doppler on the patient using a Philips^®^ high-resolution device with 5–10 MHz linear converter frequency; measurement of common carotid artery, internal carotid artery, and carotid bulb. Carotid IMT was measured at each of these segments, and patients with subclinical carotid AT were considered to have an average IMT greater than 75% [6]. (8) ABI values were measured by one-way Doppler [8].

### 2.3. Statistical Analysis

Software statistics were performed using SPSS16.0, and measurement data were reported as ($\bar{x}$ ± s). The mean ratio between the two groups was contrasted using the *t*-test. *p* < 0.05 was regarded as statistically significant when the association was examined using Person and Spearman correlation analysis.

## 3. Result

### 3.1. General Patient Information

This research had 203 participants in total, 143 of whom were male, aged 62.5 ± 6.18 years. The exacerbated patient population had a higher BODE index than those in the non-exacerbated patient subject group (3.52 ± 1.81 vs. 2.69 ± 1.46, *p* = 0.018). More patients had diabetes (23.2% vs. 12.4%, *p* = 0.017) and hypertension (68.3% vs. 52.9%, *p* = 0.024); oral hypoglycemic (23.2% vs. 14.9%, *p* = 0.039) and inhaled steroid use rates (93.9% vs. 85.9%, *p* = 0.041) were significantly higher, and patients had significantly lower diastolic blood pressure (DBP) (81.8 ± 10.9 vs. 86.3 ± 12.2 mmHg, *p* = 0.037) and FVC% (54.9 ± 11.6 vs. 60.8 ± 13.7, *p* = 0.027, Table 1).

### 3.2. Comparison of Biochemical Indexes and Inflammatory Marker Levels between Two Groups of Patients

There were notable variations in the levels of glucose, glycated hemoglobin, and white blood cell count between the two groups. Hemoglobin and hematocrit levels were higher in non-exacerbated patients, whereas higher fibrinogen and leukocyte levels were observed in the exacerbated phenotype patient population (Table 2).

### 3.3. Comparison of the Levels of Subclinical Vascular Injury in the Two Populations

The left internal carotid artery in deteriorating patients showed significantly higher IMT compared with non-deteriorating patients, and the remaining analysis parameters were similar (Table 3). Among subjects <65 years of age, the left internal carotid IMT, mean internal carotid IMT, and mean common carotid IMT were higher in exacerbated patients compared with non-exacerbated patients, in subjects ≥65 years old, and IMT levels between the two groups did not differ significantly (Table 4).

### 3.4. Correlation between COPD and Subclinical Carotid Atherosclerosis

Multivariate adjusted analysis showed that after adjustment for COPD (measured by the BODE index) and CVR (SCORE, Framingham, SCORE-HDL, Regicor, and COPD CoRi) severity, compared with non-deteriorating patients, the worsening phenotype was associated with subclinical carotid atherosclerosis (almost 3-fold increased risk, Table 5).

## 4. Discussion

COPD is a progressive lung disease that is ranked as the third leading cause of death worldwide. It is one of the most common diseases in the elderly population in China, and the number of deaths from COPD increases sharply with age in people over 70 years of age [1]. COPD is a progressive disease with a long and persistent course, causing a heavy economic burden to the patient’s family and society, seriously affecting the patient’s physical health, mental health, and mobility, and reducing his or her quality of life [2]. Previous studies have shown that in addition to pulmonary inflammatory manifestations, COPD patients are also accompanied by other severe systemic diseases outside the lung [12], such as cardiovascular disease (CVD), skeletal muscle dysfunction, bronchial malignant lesions, metabolic syndrome, diabetes mellitus, bronchiectasis, infection, and depression. Among them, CVD is a common complication of COPD [3,4]. The mortality of patients with COPD combined with CVD is 3–4 times higher than that of patients with COPD alone, and the danger of adverse cardiovascular events (such as heart failure, arrhythmia, and myocardial infarction) is significantly increased [5]. Atherosclerosis is the main pathological basis of CVD [14,15,16]. Exacerbations are linked to an increased risk of cardiovascular disease, which may be explained by a number of processes, including beta2-blockers, oral corticosteroids, hypoxemia during episodes, and a systemic inflammatory response to viral or bacterial infection [17]. The results of this research demonstrate an almost threefold higher risk of carotid subclinical atherosclerosis in patients with a COPD exacerbation phenotype compared with non-exacerbated patients. Furthermore, the severity of COPD was greater in exacerbated patients (3.4 ± 1.6 vs. 2.6 ± 1.5, *p* = 0.018), and there was a trend for higher CVR values.

A systematic review showed [18] that IMT in COPD patients was greater than that in controls. However, the relationship between the deteriorating phenotype and subclinical carotid atherosclerosis has only been briefly examined in research. When comparing 42 individuals with COPD exacerbations to 80 patients without exacerbations, Golpe R discovered no correlation between the two conditions [19]. This differs from the results of this research, which may be due to the diverse definitions of deteriorating patients. In the research by Golpe R, subjects with at least one new respiratory symptom requiring steroids and/or antibiotics in the previous year were included as exacerbations; in the present study, exacerbated patients were defined as subjects with at least two moderate (treatment changes) or severe (hospitalization) episodes. The CVR of the exacerbated subjects who participated in this study tended to be higher; it could be explained by the higher prevalence of diabetes (23.2% vs. 12.4%, *p* = 0.017) and hypertension (68.3% vs. 52.9%, *p* = 0.024). Likewise, a higher rate of vasodilator and diuretic drug use in exacerbated patients could explain the trend toward lower DBP. Furthermore, no statistically significant change in LDL-cholesterol was observed. However, according to an observational study [20], an increase of 18 mg/dL (133.2 ± 115.4 vs. 115.6 ± 26.9 mg/dL) implies an almost 20% increased coronary risk in deteriorating patients.

Inflammatory reaction is the main pathogenesis of atherosclerosis, specifically as follows: (1) inflammatory reaction caused by vascular endothelial cell injury is the initiating factor in atherosclerosis; activation of T lymphocytes and macrophages stimulates cytokine release, increasing induced endothelial cell adhesion molecule expression, leading to the circulation of the blood cells attached to the wall and migration to the vascular intima; (2) in the inflammatory response, the expression of scavenger receptors in monocytes/macrophages is increased, lipid uptake is increased, and foam cells secrete cytokines, which promotes the development of atherosclerotic plaques, and then affects the occurrence and development of atherosclerosis; (3) CRP can inhibit the process of nitric oxide synthase, affect the function of vascular endothelial cells and the generation of plasminogen activators, damage the fibrinolytic function of endothelial cells, and then promote the rupture of atherosclerotic plaques and thrombosis [18,19,20,21]. Consistent with this, it was found in this study that patients with exacerbations had increased levels of fibrinogen and leukocytes, and tended to have greater levels of CRP, compared with non-exacerbated patients. This may also explain the decreased hemoglobin and hematocrit values observed in the phenotype.

In conclusion, this research was based on the presence of subclinical atherosclerosis as determined by the thickness of the carotid intima-media. After adjusting for COPD severity (measured by the BODE index) and global cardiovascular risk including COPD CoRi, CVR was independently associated with a worsening phenotype, and these differences were more pronounced in younger patients. This suggests that the advantages of clinical preventative measures would be greater for this population.

## Figures and Tables

**Table 1:** General data of the two groups of patients.

	Total n = 203	Deteriorated Patients n = 82	Non-Deteriorating Patients n = 121	*p*
Male %	143 (73.8%)	58 (76.0%)	41 (71.9%)	0.922
Age	62.5 ± 6.18	62.1 ± 5.79	62.7 ± 6.23	0.901
Smoking %	81 (31.8%)	32 (32.0%)	18 (31.6%)	0.863
Blood oxygen saturation (ppm)	5.03 ± 6.35	4.86 ± 5.73	5.29 ± 6.88	0.372
Dyspnea MRC score (points)	1.72 ± 0.59	1.86 ± 0.56	1.68 ± 0.62	0.653
BMI (kg/m^2^)	27.9 ± 4.84	28.3 ± 5.19	27.6 ± 4.76	0.812
SBP (mmHg)	141.5 ± 19.8	137.6 ± 18.9	143.2 ± 20.4	0.794
DBP (mmHg)	83.6 ± 11.7	81.8 ± 10.9	86.3 ± 12.2	0.037
Comorbidities (%)				
Cardiovascular disease	41 (20.2%)	17 (20.7%)	24 (19.8%)	0.869
Atrial fibrillation	19 (9.36%)	10 (12.2%)	9 (7.43%)	0.157
Hypertension	129 (63.5%)	56(68.3%)	70(57.9%)	0.024
Diabetes	34 (16.7%)	19 (23.2%)	15 (12.4%)	0.017
Hyperlipidemia	72 (35.5%)	29 (35.4%)	43 (35.5%)	0.963
Renal insufficiency	3 (1.48%)	1 (1.22%)	2 (1.65%)	0.716
Metabolic syndrome	76 (37.4) %	31 (37.8%)	45 (37.2%)	0.928
Charlson Comorbidity Index	1.62 ± 0.87	1.62 ± 0.94	1.63 ± 0.86	0.990
Treatment (%)				
Statins	72(35.5%)	32(39.0%)	40(33.1%)	0.694
ACE inhibitor	42 (20.7%)	18(22.0%)	24 (19.8%)	0.753
ARA II inhibitors	59(29.1%)	25 (30.5%)	34 (28.1%)	0.726
Calcium channel blockers	34(16.7%)	15(18.3%)	19 (15.7%)	0.741
Beta blockers	14 (6.90%)	6(7.32%)	8(6.61%)	0.803
Diuretics	76 (37.4%)	34 (41.5%)	42 (34.7%)	0.425
LABA	192 (94.6%)	78 (95.1%)	114 (94.2%)	0.896
LAMA	192(94.6%)	79 (96.3%)	113(93.4%)	0.904
Inhaled steroids	181 (89.2%)	77 (93.9%)	104(85.9%)	0.041
Oral hypoglycemic drugs	37 (18.2%)	19 (23.2%)	18(14.9%)	0.039
FVC (mL)	2298 ± 752	2074 ± 619	2375 ± 823	0.926
FVC (%)	57.4 ± 12.1	54.9 ± 11.6	60.8 ± 13.7	0.027
FEV_1_ (mL)	1218 ± 435	1179 ± 398	1264 ± 506	0.895
FEV_1_ (%)	43.9 ± 14.1	41.8 ± 11.9	45.7 ± 15.7	0.713
GOLD II	66 (32.5%)	23 (28.0%)	43 (35.5%)	0.403
GOLD III	89 (43.8%)	40 (48.8%)	49 (40.5%)	
GOLD IV	36 (17.7%)	16 (19.5%)	20(16.5%)	
FEV_1_/FVC (%)	52.1 ± 10.7	53.6 ± 10.4	50.8 ± 10.9	0.826
SMWT (m)	414 ± 84.2	397.9 ± 94.3	429.4 ± 71.3	0.817
BODE Index	3.07 ± 1.58	3.52 ± 1.81	2.69 ± 1.46	0.018
CV Risk Score				
Framingham	26.4 ± 16.8	26.8 ± 16.8	25.4 ± 15.4	0.419
SCORE	7.83 ± 10.5	8.31 ± 12.4	7.52 ± 8.61	0.076
SCORE-HDL	5.58 ± 6.04	6.13 ± 6.38	5.39 ± 5.72	0.108
Regicor	6.23 ± 3.14	6.45 ± 3.37	6.01 ± 2.98	0.533
COPD CoRi	53.2 ± 9.73	53.7 ± 9.26	52.9 ± 9.83	0.812

**Table 2:** Population characteristics and inflammatory markers by phenotype.

	Total n = 203	Deteriorated Patients n = 82	Non-Deteriorating Patients n = 121	*p*
Glucose (mg/dL)	104 ± 27.9	112 ± 34.1	96.2 ± 17.6	0.014
HbA1c (%)	6.18 ± 0.9	6.29 ± 1.38	5.64 ± 0.73	0.038
Cholesterol (mg/dL)	197 ± 36.1	197 ± 42.5	198 ± 34.7	0.925
HDL (mg/dL)	57.4 ± 18.1	55.9 ± 17.7	58.8 ± 18.5	0.816
LDL (mg/dL)	123.8 ± 81.3	133.2 ± 115.4	115.6 ± 26.9	0.731
Triglycerides (mg/dL)	120.7 ± 62.5	121.2 ± 52.4	120.2 ± 70.7	0.928
Creatinine (mg/dL)	0.94 ± 0.58	1.03 ± 0.81	0.86 ± 0.32	0.714
GFR (mL)	83.5 ± 11.5	83.2 ± 11.6	83.8 ± 11.6	0.923
Uric acid (mg/dL)	7.39 ± 13.3	6.28 ± 1.96	8.15 ± 10.2	0.134
Urea (mg/dL)	38.0 ± 11.0	39.3 ± 12.5	36.8 ± 9.4	0.703
Bilirubin (mg/dL)	0.63 ± 0.27	0.62 ± 0.21	0.63 ± 0.32	0.936
GGT (U/L)	49.4 ± 82.6	51.0 ± 84.3	47.9 ± 81.9	0.618
AST (U/L)	22.8 ± 8.91	21.5 ± 7.34	24.0 ± 10.2	0.799
ALT (U/L)	23.3 ± 10.8	22.7 ± 11.5	23.9 ± 10.2	0.896
Protein (g/dL)	7.18 ± 0.54	7.21 ± 0.52	7.16 ± 0.55	0.752
TSH (μUI/mL)	2.63 ± 1.78	2.51 ± 1.82	2.72 ± 2.04	0.731
Immunoglobulin E (IU/mL)	243 ± 501	209 ± 474	274 ± 529	0.895
Hemoglobin (g/dL)	14.5 ± 1.51	14.1 ± 1.62	14.9 ± 1.43	0.007
Hematocrit (%)	45.4 ± 4.66	44.3 ± 4.82	46.3 ± 4.24	0.020
Leukocyte (10^9^/L)	7.91 ± 2.34	8.66 ± 2.17	7.35 ± 2.11	0.036
Prothrombin time (%)	100.6 ± 23.0	99.1 ± 24.4	102.0 ± 21.7	0.785
Fibrinogen (mg/dL)	415 ± 103	439 ± 98.5	394 ± 106	0.017
IL-6 (pg/mL)	5.63 ± 7.1	6.15 ± 7.04	5.03 ± 6.98	0.014
CRP (mg/L)	6.82 ± 5.2	7.52 ± 5.71	6.02 ± 4.67	0.013
TNF-α (pg/mL)	2.65 ± 0.8	2.64 ± 0.73	2.66 ± 0.92	0.879
Serum albumin (g/dL)	4.57 ± 0.3	4.43 ± 0.37	4.51 ± 0.37	0.912
Alpha-1-antitrypsin (mg/dL)	143 ± 28	144.1 ± 29.4	142.7 ± 26.9	0.962

**Table 3:** Presence of subclinical vascular injury.

	Total n = 203	Deteriorating Patients n = 82	Non-Deteriorating Patients n = 121	*p*
Right common carotid artery IMT(mm)	0.71 ± 0.13	0.72 ± 0.15	0.70 ± 0.12	0.935
Left common carotid artery IMT (mm)	0.75 ± 0.15	0.76 ± 0.15	0.73 ± 0.15	0.928
Right common carotid artery IMT (mm)	0.74 ± 0.13	0.76 ± 0.14	0.73 ± 0.12	0.906
Left common carotid artery IMT (mm)	0.75 ± 0.15	0.78 ± 0.16	0.73 ± 0.14	0.037
Right common carotid artery IMT (mm)	0.73 ± 0.14	0.73 ± 0.15	0.73 ± 0.14	0.993
Left common carotid artery IMT (mm)	0.75 ± 0.15	0.76 ± 0.15	0.73 ± 0.15	0.912
Mean common carotid artery IMT (mm)	0.73 ± 0.13	0.74 ± 0.14	0.71 ± 0.12	0.907
Mean common carotid artery ICA (mm)	0.75 ± 0.13	0.77 ± 0.14	0.73 ± 0.12	0.825
Mean common carotid artery IMT (mm)	0.74 ± 0.14	0.75 ± 0.14	0.73 ± 0.13	0.879
Plaque (%)	32(15.8%)	14(17.1%)	18(14.8%)	0.796
ABI				
≤0.9	46(22.7%)	21(25.6%)	25(20.6%)	0.298
0.9–1.3	129 (63.5%)	53(64.6%)	76(62.8%)	
>1.3	28(13.8%)	8(9.76%)	20(16.5%)	

**Table 4:** Subclinical atherosclerosis by median age and phenotype.

	≤65 Years Old			>65 Years Old		
	Deteriorating Patients	Non-Deteriorating Patients	*p*	Deteriorating Patients	Non-Deteriorating Patients	*p*
Left common carotid artery IMT (mm)	0.77 ± 0.14	0.72 ± 0.16	0.794	0.75 ± 0.15	0.74 ± 0.13	0.926
Right common carotid artery IMT (mm)	0.77 ± 0.12	0.71 ± 0.13	0.713	0.74 ± 0.17	0.74 ± 0.12	0.993
Left common carotid artery IMT (mm)	0.80 ± 0.16	0.70 ± 0.17	0.027	0.76 ± 0.15	0.74 ± 0.11	0.895
Right common carotid artery IMT (mm)	0.79 ± 0.15	0.71 ± 0.17	0.598	0.73 ± 0.15	0.75 ± 0.12	0.812
Mean internal carotid artery IMT (mm)	0.78 ± 0.13	0.71 ± 0.14	0.039	0.75 ± 0.15	0.74 ± 0.10	0.963
Mean common carotid IMT (mm)	0.77 ± 0.13	0.71 ± 0.13	0.041	0.74 ± 0.14	0.73 ± 0.10	0.928
Mean carotid bulb IMT (mm)	0.77 ± 0.13	0.71 ± 0.13	0.672	0.73 ± 0.14	0.73 ± 0.11	0.981

**Table 5:** Multivariate analysis of the association between COPD and subclinical carotid atherosclerosis.

	OR (Odds Ratio 95% CI)	*p*
Worsening phenotype	2.89 (1.08–7.63)	0.035
BODE	0.88 (0.65–1.19)	0.399
SCORE	1.02 (0.97–1.06)	0.349
Worsening phenotype	2.84 (1.09–7.59)	0.038
BODE	0.89 (0.66–1.26)	0.488
Framingham	1.04 (0.93–1.09)	0.140
Worsening phenotype	2.83 (1.08–7.56)	0.036
BODE	0.88(0.64–1.18)	0.406
SCORE-HDL	1.03 (0.96–1.09)	0.363
Worsening phenotype	2.85 (1.07–7.53)	0.035
BODE	0.88 (0.65–1.17)	0.390
REGICOR	1.06 (0.91–1.21)	0.375
Worsening phenotype	3.11 (1.141–8.46)	0.026
BODE	0.84 (0.62–1.14)	0.269
COPD CoRi	1.04 (1.04–1.21)	0.032

## Data Availability

Not applicable.
